# Clinical Experiments, Histories, and Dissections

**Published:** 1781-01

**Authors:** 


### LONDON MEDICAL JOURNAL,

### For JANUARY 1781.

### SECT. I.

### BOOKS.

I. *Clinical Experiments, Hiſtories, and Diſſections. By* Francis Home, M. D. *one of his Majeſty's Phyſicians, Fellow of the Royal College of Phyſicians of Edinburgh, and Profeſſor of Materia Medica in the Univerſity of Edinburgh.* 8*vo.* Creech, *Edinburgh*; Murray, *London.* 1780. 6s. *in boards.*

THE great advantages derived by medical ſtudents at Edinburgh from the Clinical Ward of the Infirmary, have long been deſervedly acknowledged. The beſt marked diſeaſes, ſuch as are the moſt ſingular in their nature, and the greateſt variety of acute as well as chronic complaints, are ſelected for it. Regular and circumſtantial reports of every ſymptom belonging to the diſeaſe, and of every effect produced by the remedies adminiſtered, are taken conſtantly once, and in acute and urgent caſes, generally twice a day, in preſence of the ſtudents. Theſe reports are all reviewed, when the caſe becomes the ſubject of a clinical lecture, which is delivered twice a week during the winter and ſummer ſeſſions. The utility of ſuch an inſtitution, as an appendage to a medical college, muſt be ſufficiently obvious; but its advantages are not confined merely to the ſtudent; in the hands of an inquiſitive and accurate obſerver it muſt prove a fruitful ſource of information, which may ultimately tend to the improvement of the art itſelf. This is the opinion of Dr. Home; and this opinion, we are told, produced the publication before us, which is the reſult of his attendance on the Clinical Ward during two ſummers, and a part of ſix winters.

The work is divided into twenty-four ſections. In the firſt ſection we meet with experiments to aſcertain the moſt proper time of giving the bark in intermittents. Our author obſerves, that hitherto it has been given at three different periods, periods, juſt before the fit, juſt after the fit, and from the end of one fit to the beginning of the ſucceeding one at proper intervals, but never during the paroxyſm. When it was firſt introduced, ʒij were given two hours before the fit; but Sydenham, Torti, and Cleghorn think it has not ſo good an effect when given before as after the paroxyſm. Fourteen experiments are related, from which the author concludes, 1ſt. That the bark is more efficacious in ſtopping the paroxyſm of intermittents, and curing them when given at the end of a fit, or at forty hours diſtance from the ſucceeding fit, than two, three, or four hours before it; becauſe in eight of the fourteen patients it was given juſt before the fit, but without preventing it in any one of them; whereas in five others, it was adminiſtered after the fit, and ſucceeded in all; ſo that he is perſuaded that the ſame quantity of bark (of which half an ounce was generally uſed) will, when taken towards the end of the ſweating, cure an intermittent; but will not, if given from one to four hours before the cold ſtage. 2dly, That bark given a few hours before the fit, ſeems to add to its ſeverity, from its being liable, eſpecially when given in powder, to lie long in the ſtomach. 3dly, That as bark, when given at a greater diſtance from the fit becomes more ſuccefsful, it follows, that ſome conſiderable time is required for its operation. 4thly, That the length of time before its effects appear, makes it highly probable that its operation is not on the ſtomach or on the nerves of that viſcous alone, but that it muſt enter the vaſcular ſyſtem, and there perform its chief effects. 5thly, That there appears to be no difference in the effects of the bark given after the fit, whether the ague is quotidian or tertian. With regard to theſe conclusions, we beg leave to obſerve, that we have often given half an ounce of the bark within two hours of the expected return of the paroxyſm. In ſome inſtances it failed; but in others, and in the greater number, it prevented the fit. We never obſerved, that it added to the ſeverity of the paroxyſm. We are perſuaded however from repeated obſervations, that the beſt method of adminiſtering this remedy is from the end of one fit to the beginning of the next.

In the ſecond ſection the author relates his experiments in the typhus nervoſus, or low fever, as he terms it, which it ſeems is by much the moſt frequent at Edinburgh, where a pure ſynocha nocha is ſeldom ſeen, even amongſt the commonalty. In ſome caſes of this ſort our author tried the bark; in one of theſe, which was accompanied with a difficulty of breathing, it ſeemed to do harm; in the other three he thought it of uſe. When there is general ſymptomatic ſweating, tremor, or little drought, he thinks it may be preſcribed to advantage; its effects, in moſt points, being ſimilar to thoſe of wine, with this difference however, that they appear later and are of longer duration than the latter. In five other caſes our author tried the tinctura cantharides, which he conſiders as an innocent remedy when taken to gut. xx thrice a day; the only ſenſible effect it produced being a ſenſation of heat in the ſtomach, except in one patient in whom it occaſioned gripes. By combining it with mucilage of gum arabic he has been enabled to increaſe the doſe of it to gut. xxxv. four times a day in cutaneous diſeaſes; and to gut. l. in a diabetes, without any viſible effects on the urinary paſſages. From ſeven caſes of typhus, in which bliſters were applied at a time when no other remedy was employed, he concludes them to be of ſmall advantage in this diſeaſe; five of the ſeven patients not having been ſenſibly relieved lieved by them; and their good effects, in the two others, having appeared doubtful: our author endeavours to account for this by obferving, that as the ftimulating power of blifters lafts only two or three hours, and of courfe too fhort a time to be of much fervice, their antifpafmodic effects, which take place aftet this ftimulus is over, will do more harm than the latter has done good. Dr. Home has perhaps been too hafty in his conclufion here, as we have often experienced the moft excellent effects from blifters in cafes of typhus. He recommends blifters to the temples in this difeafe, not with a view to produce any good effects on the fever, but merely to relieve the head-ach, and in this way we are told he has often experienced their efficacy. We wifh we could always prevent the ftrangury, which is fo frequently an effect of thefe applications. Our author afferts that it may eafily be done by fprinkling camphor on the blifter; and fo Dr. Greenfield afferted long ago, but we fear without fufficient reafon. We have feen ftrangury produced in a violent degree, though camphor was rubbed upon the blifter, and at the fame time given internally. The effects of fomentation of the legs are next related. Our author author tried this method in ſeven caſes, and found it produced a ſtronger pulſe, a diſpoſition to ſleep, and a moiſture of the ſkin. As its good effects depend upon its ſtimulus, he advifes the heat to be above 100° of Fahr. Therm. becauſe if it is within this, it will ſtimulate leſs, and relax more. Dr. Home gave camphor in five caſes, but he is dubious with regard to its effects; and reſerves the deciſion for further experiments. From its ſedative properties he ſeems diſpoſed to think rather unfavourably of it in typhus; but we think it right to obſerve here, that the ſedative powers of this ſubſtance have not yet been ſatisfactorily aſcertained; and our author cannot but know that Sir John Pringle, who experienced its good effects in malignant fevers, aſcribes them to its antiſeptic properties. The author next preſents us with a comparative view of the effects of emetic tartar and James's powder in ſeventeen caſes of typhus; he gives the preference to the latter, from his having found it to be much gentler in its operation than the other, and from its commonly producing a calmer ſtate, and ſleep, which the emetic tartar ſeldom does. The latter, our author adds, acts more on the inteſtinal canal; James's powder more more on the ſkin. If we wiſh to make a ſudden and violent ſtimulus on all the organs of evacuation, we ſhould uſe the tartar emetic; but in delirium, want of ſleep, looſe or irritable belly, James's powder, he contends, ought to be preferred. In eight caſes of typhus Dr. Home gave from xx to xxv drops of the thebaic tincture at bed-time, and found that it procured ſleep without producing any ill effect. Petaſites or butter-bur, which had been uſed in Muſcovy in a malignant fever that prevailed there ſome years ago, was tried by our author in one caſe, but it produced no ſenſible effects, though given in doſes of a drachm three times a day.

The ſubject of the third ſection is a ſpurious kind of pleuriſy that prevailed in the Clinical Ward in December 1776; the characteriſtic ſymptoms of this diſeaſe were a painful ſtitch, conſtant dry cough, ſevere head-ach, difficult breathing, quick and weak pulſe. As this complaint was ſhort, and not attended with danger, the alleviation of the ſymptoms claimed moſt of the author's attention.

In the fourth ſection Dr. Home relates two hiſtories of puerperal fever. One of the patients recovered; the other died on the ſixth day.

After

After entering very fully into the cauſes and treatment of this diſeaſe as it has occurred to himſelf and others, our author concludes with obſerving that we know little of the nature, and ſtill leſs of the cure of the puerperal fever; and that our chief aim ſhould be to prevent it, which may probably be done by a proper ventilation of the rooms, by not allowing the curtains to be ſhut, by prohibiting all fire and load of bedcloaths, by giving cool drink, by ſhunning all animal food, unleſs in nervous habits, and by avoiding, after delivery, all ſtrait binding over the belly.

In the fifth ſection the author gives a caſe of meaſles that proved fatal on the fifteenth day. The patient was a female, aged twenty-four. Upon diſſection the trachea was found filled with matter of a purulent appearance.

Next follow experiments upon ſome remedies uſed in the phthiſis pulmonalis. In three caſes the vitriolic acid was tried, but did not produce any good effect. It conſtantly excited purging. In one of theſe patients from ten to fifteen grains of alum were given to check the diarrhœa, but he was no better, and his pulſe ſeemed quicker while he took it. The

ſame patient afterwards took ʒſs. of the bark four times a day for fifteen days, at the end of which time all the ſymptoms were evidently worſe. During nine other days our author gave him ſtarch, but without much advantage. Four experiments are related to prove that the inhaling of mephitic air may ſometimes be advantageous; but the author very properly obſerves, that more trials are neceſſary to determine its effects in this diſeaſe, and the degree of truſt it deſerves. In two patients he tried the vapour of Thus, but he gave it up as being too irritating.

The ſeventh ſection is employed on the melæne, which is the νȣσος μελαινης, or *Morbus niger* of Hippocrates. The black ſtools which characterize this diſeaſe, and which the ancients and ſome of the moderns have aſcribed to the atra bilis, ſeem to be merely owing to an effuſion of blood into the inteſtinal canal. Dr. Cullen, in his Noſology, has very properly omitted to conſider it as a diſtinct diſeaſe. Our author found gentle laxatives, and vitriolic acid combined with gum arabic, of uſe in this complaint.

In the eighth ſection Dr. Home deſcribes his experiments upon the *rhododendron chryſanthemum*, Linn. which has been much uſed in Siberia as a remedy medy for the rheumatiſm. It is aſtringent, and appears to be powerfully ſedative. It is given in infuſion from ℥ſs. to ℥iij. Our author tried it only in three caſes, and from the little ſucceſs that attended it in theſe, concludes it to be much inferior, in its effects, to many other remedies that are uſed in rheumatiſm. In one of the caſes the diſeaſe was next day cured by pulv. dover.

The ninth ſection is allotted to a caſe of cephalalgia that terminated fatally. On diſſection much water was found in the ventricles and around the medulla oblongata, hydatids adhering to the plexus choroides, and an effuſion of blood between the pia mater and tunica arachnoides near the falx in the left hemiſphere.

In the tenth ſection we meet with two caſes of albugo or leucoma, which the author thinks were cured by Sir Hans Sloane's ointment. This concluſion, however, does not ſeem to be warrantable, as bleeding, laxatives, bliſters, &c. were employed in both patients.

The next ſection, which is a very long one, is employed on antiſpaſmodics. The firſt in the liſt is *Fear*. One caſe is related of an hyſteric woman, who, in conſequence of a fright, was relieved from her ſymptoms for five days, but they then returned. 2d. The cold bath—Our author tried this in one patient who had a convulſion of the whole left ſide, but it brought on a ſevere fit. 3d. Venæ ſectio—In two caſes of hyſteria from Amenorrhœa it ſeemed to be uſeful. In two caſes of aſthma it was of no uſe, but in two others the ſymptoms were alleviated by it. In an epileptic boy V. S. ſeemed to do harm. In three caſes of ſingultus (which our author conſiders as idiopathic, though to us they ſeem to have been merely ſymptomatic from hyſteria) it was beneficial. Here we cannot avoid taking notice again of the ſtrange concluſions Dr. Home ſometimes deduces from his caſes; becauſe theſe *three* patients with ſingultus happened to be women, he ſuppoſes that ſex to be the moſt frequently ſubject to this affection; and becauſe *two* of the *three* were unmarried, he concludes that umarried women muſt be the moſt liable to it. 4th. Electricity—The author certainly did not give this remedy a fair trial: in one caſe (*ſingultus*) it was uſed only four, and in another (*tremor palpitans* Sauv.) only five days. 5th. Bliſters.—Nine caſes are related, in which bliſters were applied, and the author's concluſion from theſe is, is, that they did not ſeem to be powerful antiſpaſmodics. But we muſt own that ſeveral of the caſes as he has related them ſeem to prove juſt the contrary. In the firſt caſe, for inſtance (a ſingultus in a young woman of eighteen, which had reſiſted a variety of remedies) a bliſter was applied near the falſe ribs in the direction of the diaphragm, Aug. 1, and the hiccup appeared no more, adds the doctor, excepting on the 4th; on the 6th ſhe was diſmiſſed, cured. In the ſecond patient (another caſe of ſingultus) a bliſter applied towards the latter end of the diſeaſe relieved her for three days. The ſame effect was produced in the third patient; though the hiccup returned in both caſes after that time. In another it removed the pain, though it did not alleviate the convulſive breathing. In the other caſes, which were aſthmatic, bliſters do not ſeem to have had a fair trial. We have generally found them very ſerviceable in the ſpaſmodic aſthma. 6. Valerian—Nine caſes are related of ſpaſmodic affections in which this remedy was given without much ſucceſs, as it was of uſe only in one patient, a woman of fifty-three, who had been for two months troubled with vertigo and palpitations. It is obſervable however, that that this woman was the only one who continued its uſe more than ten days. She perſevered in it for eleven days, and took ℥jſs. four times a day. The other patients took only from ℈ij to ℥j, and it was diſcontinued after ſix days, except in one woman who uſed it during ten days. We have never ſeen any good effects from this ſubſtance, unleſs it was given in large and frequently repeated doſes. 7th. Muſk—In ſix caſes, purely ſpaſmodic, from ten to fifteen grains of this ſubſtance were given twice or thrice a day without any good effect. Larger doſes would perhaps have proved more efficacious. 8th. Camphire—From the effects of this remedy in ſix trials Dr. Home conſiders it as a more powerful antiſpaſmodic than muſk; as it makes the pulſe ſlower, and rather cools, he thinks it is chiefly indicated in inflammatory ſpaſms; but he finds that its effects are not of long duration; as the diſeaſe is apt to return. He generally exhibited ten grains of it three times a day. 9th. Caſtor—This was given without any effect in three ſpaſmodic caſes, but in ſpaſmodic feveriſh caſes our author has found it uſeful, as it makes the pulſe ſlower, and acts as a ſedative. 10th. Aſſafœtida—Dr. Home thinks this medicine poſſeſſes poſſeſſes conſiderable antiſpaſmodic powers, but as it heats and quickens the pulſe, he cautions us againſt its uſe in inflammatory caſes. 11th. Spiritus æthereus vitriolicus—This medicine ſeemed to be of uſe in the hyſteria; of eight patients it cured three, and relieved two: Dr. Home obſerves, that its good effects are not increaſed by augmenting the doſe, as he ſucceeded with a teaſpoonful twice or three times a day, while the largeſt doſes were ineffectual. 12th. Cortex Peruvianus—Our author ſpeaks of this as an excellent remedy in pure ſpaſmodic diſeaſes. He ſucceeded with it in ſeven caſes, which were of the anti-inflammatory kind. 13th. Artemiſia—This was tried only in one caſe, and it ſucceeded after aſſafœtida and æther had been given without any effect. A drachm of the leaves in powder was exhibited four times a day. 14th. Pæonia—The root of this plant, which was much employed by the antients as an antiepileptic, was given in two caſes in doſes of ʒ ſs four times a day without effect. 15th. Viſcus quercinus, or miſletoe, was employed in another caſe of epilepſy, but with no better ſucceſs. Our author ſpeaks equally unfavourably of the extractum hyoſcyami, folia aurantiorum & flores cardamine pratenſis, pratensis, neither of which he thinks possess any antispasmodic properties. 16th. Opium—This remedy seemed to do no good in a case of epilepsy, and another of convulsio, but in two cases of hysteria he found it very efficacious, as it cured both patients; and in a case of asthma it afforded much relief to the patient. 17th. Cuprum ammoniacale—This was tried in four cases, but without any other success than its suspending the fits for a few days in one patient. 18th. Flores zinci—Since Gaubius's detection of the quack remedy used by Ludemanus, this medicine has been much employed in epileptic and other spasmodic affections. Dr. Home tried it in four cases of epilepsy, two of asthma, one of hemitotonos, and two of hysteria. It was useful in the epileptic, but of little or no service in the other cases. He began with it in a small dose, which he gradually increased in some of the patients to ℨss. twice a day. It generally produced a nausea, rarely purged, sometimes sweated, and often had no sensible effect. 19th. Mercury—This remedy, which was first recommended as an antispasmodic in the *Essays & Obs. Phys. & Lit.* succeeded in the hands of our author in two two remarkable caſes, a triſmus clonicus, and ſpaſmus gulæ, the latter of which ſeemed to yield to no other antiſpaſmodic. But it failed twice in a tremor palpitans, two aſthmas, an hyſteria, and hemitotonos. Our author concludes the ſection with the following obſervations, which we ſhall give in his own words:

"It is but a melancholy retroſpect to view ſo "many trials made with the moſt approved an-"tiſpaſmodics, and ſo few cures performed by "any one particular remedy. We ſee that there "is no ſpecific in which we can always truſt, "but muſt vary our medicines, as a new one "will often ſucceed, when others have before "failed. This uncertainty of antiſpaſmodics de-"pends not, perhaps, ſo much on the ſtubborn "nature of ſuch diſeaſes, or on the weakneſs of "the remedies, as on the want of accurate expe-"riments, with all their circumſtances. This "has been a great defect in the materia medica, "has ſtopt the progreſs of medicine, and kept "it in a ſtate of uncertainty; whereas if the "circumſtances of the diſeaſe, and of the ex-"hibition of the remedy, had been handed "down, certain and fixed general principles and "rules muſt, ere this time, have taken place.

" To ſupply this defect, and point out the pro-

" per line for the improvement of medicine, I

" have collected the preceding experiments.

" Antiſpaſmodics are not all entitled to equal

" confidence. I know no author, however, who

" has ſettled their comparative merit. Each

" phyſician is left to judge from his own ex-

" perience. But in private practice he may

" grow old without facts ſufficient. Were I,

" from the preceding experiments, which are

" not few, to make a computation of their com-

" parative value, I would arrange them into

" four claſſes, according to their powers. In

" the firſt I would place the weakeſt, as fol.

" aurant. flor. cardam. artemiſia, peonia, viſcus

" quercinus, extr. hyoſcyam. caſtor, muſk,

" cuprum ammon. electricity. In the ſecond,

" fear, camphire, flor. zinci, bliſters. In the

" the third, aſſ. fœtid. æther, mercury. In the

" fourth, bark, opium, bleeding. Every one,

" in the diſtribution, will judge as he has ex-

" perienced. I may alter my opinion on further

" trials, as it is from theſe I have formed the

" preſent. It is good, however, to have ſome-

" thing fixed, as it is eaſier afterwards to correct

" than to ſettle at firſt ſuch a comparative view.

" One

" One of the chief defigns of thefe experiments " was to difcover the cafes and fituation in which " fuch medicines might be moft fuccefsfully " ufed. In this we have not been altogether un- " fuccefsful. We may obferve that moft of " thefe, befides their primary antifpafmodic qua- " lity, poffefs fecondary qualities, which have " much influence in their effects and exhibitions. " Befides fome of them poffeffing laxative or " fudorific powers, which others do not, they " may be diftinguifhed into the ftimulant, or " inflammatory, and fedative, or anti-inflamma- " tory. Of the former fort, are electricity, " mercury, valerian, affafœtida, cortex Peruvi- " anus, opium, &c. Of the latter, are bleed- " ing, epifpaftics, mufk, camphire, caftor, " æther, flor. cardamin. fol. aurant. cupr. " ammon. flor. zinci, &c. The former muft " be chiefly ufeful in the debile anti-inflamma- " tory ftates; the latter, in the febrile and in- " flammatory. The preceding experiments have " confirmed this: and bleeding has been found " one of the moft powerful anti-hyfterics, when " the ftate was inflammatory.

" But particular antifpafmodics are fuited to " cure particular fpafmodic difeafes from fome

" other circumstances, independent of those just

" now mentioned. These experiments have

" shewn me the fact, but they have not disco-

" vered the cause or principle on which it de-

" pends. Æther will relieve one spasmodic

" disease, and not another, though both inflam-

" matory, or both anti-inflammatory. Flor.

" zinci will cure an epilepsy, though not a

" convulsio. Opium will ease an asthma, though

" not a convulsio. Mercury will cure a tris-

" mus or spasmus gulæ, though not an hysteria,

" convulsio, or asthma."

[ *To be concluded in our next.* ]

