# Errata

**Published:** 1781-06

**Authors:** 


					ERRATA.
Page?io, line 35, for chyrfanthemum read chryfanthum.-

				

